# Designing Sustainable Hydrophilic Interfaces via Feature Selection from Molecular Descriptors and Time-Domain Nuclear Magnetic Resonance Relaxation Curves

**DOI:** 10.3390/polym16060824

**Published:** 2024-03-15

**Authors:** Masayuki Okada, Yoshifumi Amamoto, Jun Kikuchi

**Affiliations:** 1 Graduate School of Bioagricultural Sciences, Nagoya University, 1 Furo-cho, Chikusa-ku, Nagoya 464-8601, Japan; masayuki.okada@a.riken.jp; 2 RIKEN Center for Sustainable Resource Science, 1-7-22 Suehiro-cho, Tsurumi-ku, Yokohama 230-0045, Japan; yoshifumi.amamoto@a.riken.jp; 3 Graduate School of Social Data Science, Hitotsubashi University, 2-1 Naka, Kunitachi 186-8601, Japan; 4 Graduate School of Medical Life Science, Yokohama City University, 1-7-29 Suehiro-cho, Tsurumi-ku, Yokohama 230-0045, Japan

**Keywords:** hydrophilic coating materials, time-domain nuclear magnetic resonance, contact angle, chemoinformatics descriptors, machine learning

## Abstract

Surface modification using hydrophilic polymer coatings is a sustainable approach for preventing membrane clogging due to foulant adhesion to water treatment membranes and reducing membrane-replacement frequency. Typically, both molecular descriptors and time-domain nuclear magnetic resonance (TD-NMR) data, which reveal physicochemical properties and polymer-chain dynamics, respectively, are required to predict the properties and understand the mechanisms of hydrophilic polymer coatings. However, studies on the selection of essential components from high-dimensional data and their application to the prediction of surface properties are scarce. Therefore, we developed a method for selecting features from combined high-dimensional molecular descriptors and TD-NMR data. The molecular descriptors of the monomers present in polyethylene terephthalate films were calculated using RDKit, an open-source chemoinformatics toolkit, and TD-NMR spectroscopy was performed over a wide time range using five-pulse sequences to investigate the mobility of the polymer chains. The model that analyzed the data using the random forest algorithm, after reducing the features using gradient boosting machine-based recursive feature elimination, achieved the highest prediction accuracy. The proposed method enables the extraction of important elements from both descriptors of surface properties and can contribute to the development of new sustainable materials and material-specific informatics methodologies encompassing multiple information modalities.

## 1. Introduction

The bioeconomy [1] and circular economy [2] are the keys to realizing a sustainable society. With the shift in focus toward the management of the life cycle of plastics [3], understanding the interfaces of materials has become crucial. For example, understanding the mechanisms underlying biological and chemical reactions, including microorganism reactions that degrade polyethylene, polystyrene, and polypropylene [4,5], enzyme reactions that degrade polyethylene terephthalate (PET) [6], and marine biofouling, which occurs at the interfaces of materials, is crucial [7]. Therefore, methods based on wettability, which indicates the hydrophilicity and hydrophobicity of a material, antifoulant release, self-renewability, temperature and pH changes, and biomimetics have been developed [8]. Biocompatible 2-methacryloyloxyethyl phosphorylcholine, developed by introducing phosphatidycholine, which is a component present in biological membranes [9] has a hydration layer formed on the polymer imparts strong antifouling properties [10]. A hydration layer, namely an intermediate water layer, formed on poly(2-methoxyethyl acrylate) (PMEA) plays a critical role in preventing fouling [11,12]. Thus, polymer hydrophilicity as well as hydrophobic coatings play crucial roles in controlling fouling. Superhydrophobic polymers exhibit antifouling properties owing to their low surface free energies [13]. In addition, elastomers based on silicone or polydimethylsiloxane are used to prevent fouling. However, the adhesion between the coating material and substrates is weak, and thus, various studies have been conducted to improve the adhesion using nanofiller mixtures [14].

The functionality of polymers are influenced by their microscopic molecular structures as well as their intricate molecular dynamics, including the behavior of polymer chains and their entanglement. Hence, comprehending and managing molecular dynamics is pivotal for effectively understanding and harnessing polymer properties [15]. The surface properties of materials, such as rigidity, affect their hydrophilicity [16]. Nuclear magnetic resonance (NMR) is a powerful tool for analyzing molecular dynamics, and the corresponding signals can be measured over a wide range of timescales, from picoseconds to milliseconds (ms) [17]. Thus, NMR spectroscopy can be employed to study the relationships between the functions and physical properties of materials and their structures [18]. Proton and carbon-13 nuclear magnetic resonance (^1^H NMR and ^13^C NMR) spectroscopies are frequently used in polymer development [18,19], and time-domain NMR (TD-NMR), a highly promising analytical tool, is extensively utilized to explore the impact of internal and external factors on the structure and properties of various materials, including polymers, fresh foods, processed food products, and agricultural items [20].

Because NMR spectroscopy generates a large amount of high-dimensional data pertaining to molecular dynamics, various measurement informatics technologies have been simultaneously developed to streamline the associated measurement process [21]. To optimize machine learning (ML) performance, the extraction of optimal features from raw data is crucial. Moreover, high-dimensional datasets often lead to overfitting issues [22]. To address these challenges, approaches involving dimensionality reduction and/or feature selection are employed. Methods such as principal component analysis [23], multidimensional scaling [24], and linear discriminant analysis [25] are used for reducing feature space dimensions owing to their high efficacy in identifying highly relevant descriptors, which are commonly referred to as key features and are particularly beneficial for ML applications [26]. Additionally, non-negative matrix factorization (NMF), partial least squares [27], and semi-supervised NMF have been used as dimension reduction methods [28], in which genetic algorithms [29,30] are utilized to meaningfully reduce the relaxation component to 10% [31]. Recursive feature elimination (RFE), a type of feature selection, is applied to perform quality control of polylactic acid processing [32] and antibacterial peptide development [33]. Recently, a new RFE approach that evaluates the “feature (variable) importance” based on support vector machine (SVM), random forest (RF), and gradient boosting machine (GBM) models as well as selects and eliminates the least important features has been proposed [34]. 

As mentioned before, analyzing microscopic molecular structures is also necessary for assessing the surface properties of materials. For instance, in an antifouling membrane [8], after the initial formation of conditioning films, microorganism adhesion occurs [35]. The antifouling ability of PMEA originates from the interactions between the constituent carboxy and methoxy groups with water molecules [36]. Hence, controlling the intermolecular interactions is crucial. Materials informatics (MI) involves the analysis of microscopic surficial molecular structures using informatics technology. To date, various MI models based on molecular descriptors have been developed using open-source tools, such as RDKit, which is widely utilized in chemoinformatics. These models aid in devising synthesis strategies for molecules, including inorganic nickel (II) salts, organic photosensitizers [37], and amphiphilic copolymers [38]. Although MI research is primarily focused on extracting essential physicochemical components, progress in integrating these findings with molecular dynamics has been limited. Specifically, studies on the application of diverse types of RFE algorithms to NMR transition curves are scarce.

**Figure 1:**
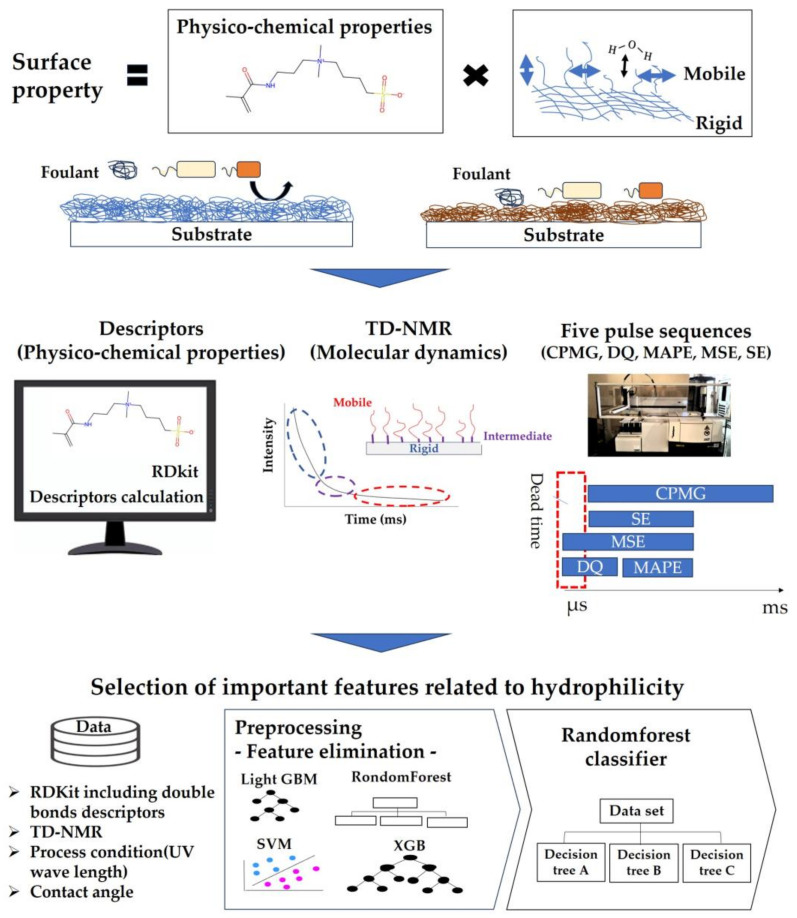
Conceptual framework of the study, illustrating the pivotal role of molecular descriptors and molecular dynamics in hydrophilicity development. The study encompassed calculations and measurements of these elements. The dataset, comprising RDKit, TD-NMR, contact angle data, and manufacturing process details, underwent feature selection via RFE. Preprocessed data were then utilized by RF classifier to identify crucial factors for building predictive models of hydrophilicity and investigating the underlying principles governing this trait.

In the present study, we constructed an ML model that incorporates both molecular and dynamics descriptors to predict the hydrophilicity of hydrophilic polymer coating materials. The conceptual framework of the study is illustrated in Figure 1. We conducted RF classification using a combination of RDKit descriptors, five distinct pulse sequences from TD-NMR spectroscopy, and different ultraviolet (UV) wavelengths applied during the manufacturing of the coating material. This study was focused on enhancing interpretability through the application of RFE as a feature selection method.

## 2. Materials and Methods

### 2.1. Sample Preparation

#### 2.1.1. Materials

*N*,*N′*-{[(2-acrylamide-2-[(3-acrylamidopropoxy) methyl] propane-1,3-diyl) bis (oxy)] bis(propane-1,3-diyl)}diacrylamide (FOM-3006), *N*,*N′*,*N″*-triacryloydiethylenetriamine (FOM-3007), *N*,*N′*-diacryloyl-4,7,10-trioxa-1,13-tridecanediamine (FOM-3008), *N*,*N′*,*N″*,*N‴*-tetraacryloyltriethylenetetramine (FOM-3009), and [3-(Methacryloylamino)propyl]dimethyl(3-sulfobutyl)ammonium hydroxide inner salt (FOM-3010), *N*-*tert*-butylacrylamide (NTBA) were used as hydrophilic monomers (Figure S1). 2-Hydroxy-4′-(2-hydroxyethoxy)-2-methylpropiophenone (Irgacure 2959) was used as the photoinitiator. Methanol was used as the solvent to dissolve the monomers. FOM-3006, FOM-3007, FOM-3008, FOM-3009, FOM-3010, and methanol were obtained from Fujifilm Wako Pure Chemical Corporation (Osaka, Japan). NTBA and Irgacure 2959 were purchased from Tokyo Chemical Industry Co., Ltd. (Tokyo, Japan) and Sigma-Aldrich (Tokyo, Japan), respectively.

#### 2.1.2. Surface Coating

Surface coatings were prepared by copolymerizing two types of hydrophilic monomers on PET sheets, which were adopted owing to the strong affinity of PET with hydrophilic polymers as well as a high recycling rate (approximately 85% in Japan) of PET [39]; owing to its high recycling rate, PET is one of the most sustainable plastics.

Two monomers were randomly selected and dissolved in methanol, which contained the photoinitiators listed in Table S1. Approximately 200 μL of each mixture was coated onto a PET sheet, with dimensions of approximately 3.5 cm × 3.5 cm (Cosmoshine A4360, TOYOBO, Osaka, Japan), using a micropipette. The mixing ratio of each monomer is listed in Table S1. The coated sheets were dried at 50 °C for 10 min using a constant temperature thermostatic dryer natural oven (NDO-420, TOKYO RIKAKIKAI CO., LTD., Tokyo, Japan). Subsequently, the sheets were exposed to UV light, with a wavelength of either 254 nm or 365 nm, inside a box using a handy UV light (SLUV-4, AS ONE CORPORATION, Osaka, Japan) for curing.

### 2.2. TD-NMR Measurements

TD-NMR measurements were conducted at 298 K using the Minispec mq20 NMR spectrometer (Bruker, Billerica, MA, USA) to assess the dynamics of the polymer chains within the hydrophilic coating. This equipment is equipped with Carr-Purcell Meiboom-Gill (CPMG) [28], double quantum (DQ) filter [40], magic sandwich echo (MSE) [40], solid echo (SE) [41], and magic and polarization echo (MAPE) [40]. The PET sheets were cut into square shapes measuring approximately 1 mm × 10 mm and placed in measurement tubes without any solvent. *T*_2_ relaxation curves were obtained using five pulse sequences, viz, CPMG, DQ, MSE, SE, and MAPE. Because CPMG was a pulse sequence that could measure long relaxation times in the order of ms, information on rapid molecular mobility was obtained. SE could measure short relaxation times in the order of μs and was thus used to measure high-order structures, such as crystalline and amorphous structures. However, the application of SE was limited by its associated dead time. Thus, MSE, DQ, and MAPE pulse sequences, which could overcome this dead time issue, were employed. MSE consisted of DQ and MAPE, and DQ could measure extremely short relaxation times, whereas MAPE could measure relaxation times longer than those measured by DQ. The relaxation curves exhibited distinct time regions for the slow and fast mobile components of the polymers. Specifically, the MAPE, DQ, MSE, and SE sequences depicted the slow mobile components, while CPMG captured the fast mobile components in ms. MSE represented both slow and relatively fast mobile components. Furthermore, the DQ and SE sequences detected the slow mobile components with short relaxation times.

### 2.3. Contact Angle Measurement

Contact angle measurements were conducted using a contact angle meter (DMs-401, Kyowa Interface Science Co., Ltd., Saitama, Japan). A 2 μL droplet of clean water was dispensed using a microsyringe, and its side image was captured using the accompanying digital camera. From the obtained image, the contact angle was automatically calculated using the *θ*/2 method. The contact angle of each specimen was measured thrice, and their average value was adopted as the final measured contact angle. Based on the measured contact angles, the films were divided into two groups: films with contact angles less than 25°, 30°, 35°, or 40° were categorized as 0, while those with contact angles exceeding 25°, 30°, 35°, or 40° were classified as 1. Such a classification was performed to avoid large differences in the amount of data after classification.

### 2.4. Generation of Molecular Descriptors

Molecular descriptors of the monomers were generated using a simplified molecular input line entry system [42], which transformed chemical structures into text representations, and RDKit (version 2023.3.2) (Table S2). The descriptors of all the monomers were selected (Table S3). The molecular descriptors of the copolymers were calculated based on the mixing ratio of the monomers and photoinitiators. Additionally, the number of chemical bonds, including double bonds (C–C, C=C, C–N, C–O), in each monomer was utilized as a descriptor. Furthermore, the bond distances between the vinyl groups were manually calculated based on individual bond numbers and bond lengths (C–C: 1.54 Å, C=C: 1.34 Å, C–N: 1.43 Å, and C–O: 1.43 Å).

### 2.5. Data Analysis

The data were processed using Python (version 3.10.12), scikit-learn library (version 1.2.2), LightGBM (version 1.2.2), and XGBoost (version 2.02). Autoscaling (standardization) was conducted for both the molecular descriptors and TD-NMR relaxation curves. Because the mobile molecules were assumed to be contributors to hydrophilicity, TD-NMR and data from five pulse sequences were employed in the analysis. Feature selection was executed using GBM-RFE, RF-RFE, SVM-RFE, and XGM-RFE. To ensure that the number of features remains less than the number of samples (=57), the number of features after reduction was set to 30. The features obtained via RFE were employed as explanatory variables, while the binary classification values of the contact angle were employed as target variables. RF classifiers were used to construct classification models. The data were split into training and test datasets using the holdout method. Hyperparameters were determined using cross-validation methods by applying GridSearchCV to the training data. Prediction accuracies were assessed based on the parameters: accuracy, precision, recall, and F1-score. The flow of data analysis is depicted in Figure S2, and the hyperparameter RF is shown in Table S4.

## 3. Results

### 3.1. Surface Coating

Surface coatings were applied by the photoinitiated copolymerization of acrylamide monomers on PET films. Both ionic (FOM-3010)) and nonionic (NBTA) monomers were utilized to alter the surface properties. Cross-linkers with varying numbers of vinyl groups were employed to stabilize the coating and regulate film dynamics. Upon exposure to UV light, the initially flowable liquid transformed into a cured solid with high viscosity. As a result, polyacrylamide was coated onto the PET films through the copolymerization of the monomers. 

### 3.2. TD-NMR

Changes in chain dynamics due to surface modifications were assessed through TD-NMR measurements. Figure S3 displays the TD-NMR relaxation curves, which are correlated with the surface modification conditions, acquired for various pulse sequences. Noticeable distinctions in the relaxation curves were evident for CPMG, MSE, and SE sequences. Specifically, the CPMG and MAPE [40] appeared suitable for mobile components, suggesting their effectiveness in detecting components characterized by long relaxation times on the surface.

**Figure 2:**
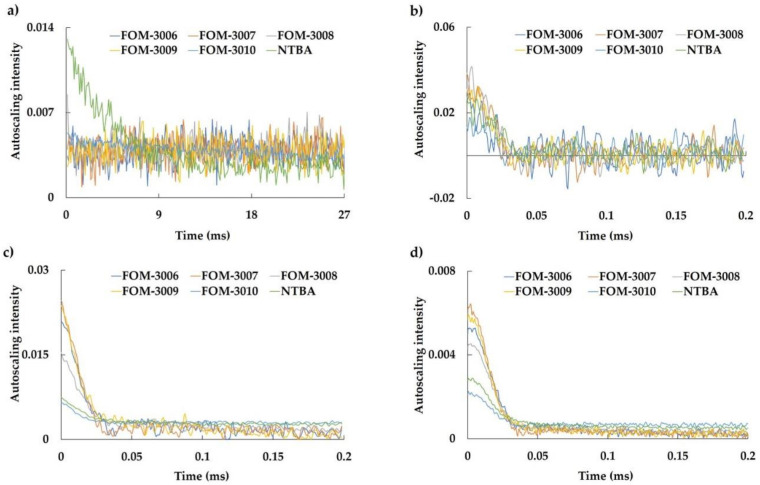
*T*_2_ relaxation curves of each single-monomer polymer. (**a**) CPMG, (**b**) DQ, (**c**) MSE, and (**d**) SE. The blue, orange, gray, yellow, and light and dark green lines represent FOM-3006, FOM-3007, FOM-3008, FOM-3009, FOM-3010, and NTBA, respectively.

The impact of the presence or absence of cross-linkers on the relaxation curves was confirmed, as depicted in Figure 2. The evaluation of samples coated with NTBA using the CPMG sequence revealed a gradual attenuation in the intensity of *T*_2_ relaxation. Conversely, the relaxation curve obtained using the MSE sequences exhibited a low intensity with minimal changes. However, for samples with cross-linkers, the CPMG relaxation curves exhibited minimal alterations, with sharper relaxations observed in the MSE sequences. These trends were accentuated with an increase in the number of vinyl groups. Polyacrylamide prepared with NTBA featuring a single vinyl group exhibited linear polymer chains, while FOM-3006, FOM-3007, FOM-3008, and FOM-3009, which possess multiple vinyl groups, displayed cross-linked or networked structures with reduced mobility. Thus, these findings underscore a disparity in the TD-NMR relaxation curves, stemming from the chain mobilities of polyacrylamide on the surface.

### 3.3. Contact Angle

**Figure 3:**
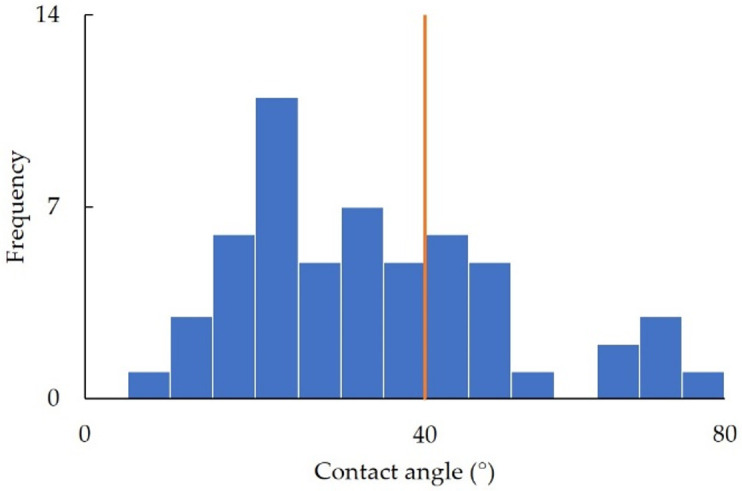
Contact angle histogram, with each bin representing a 5° interval ranging from 10° to 80°. The orange line represents 40°, which is the criteria for binary classification.

The properties of the modified surfaces were evaluated through contact angle measurements. Figure 3 illustrates a histogram of the contact angles of the sample films. The contact angles of the modified surfaces exhibited a broad range of values, spanning from 5° to 80°. To determine the standard for binary classification, data analysis was performed according to the approach indicated in Section 2.5, and the best results were obtained at 40°. Therefore, 40° was set as the criteria for binary classification. The performance data for angles of 25°, 30°, and 35° are shown in Figure S4.

### 3.4. RFE

**Figure 4:**
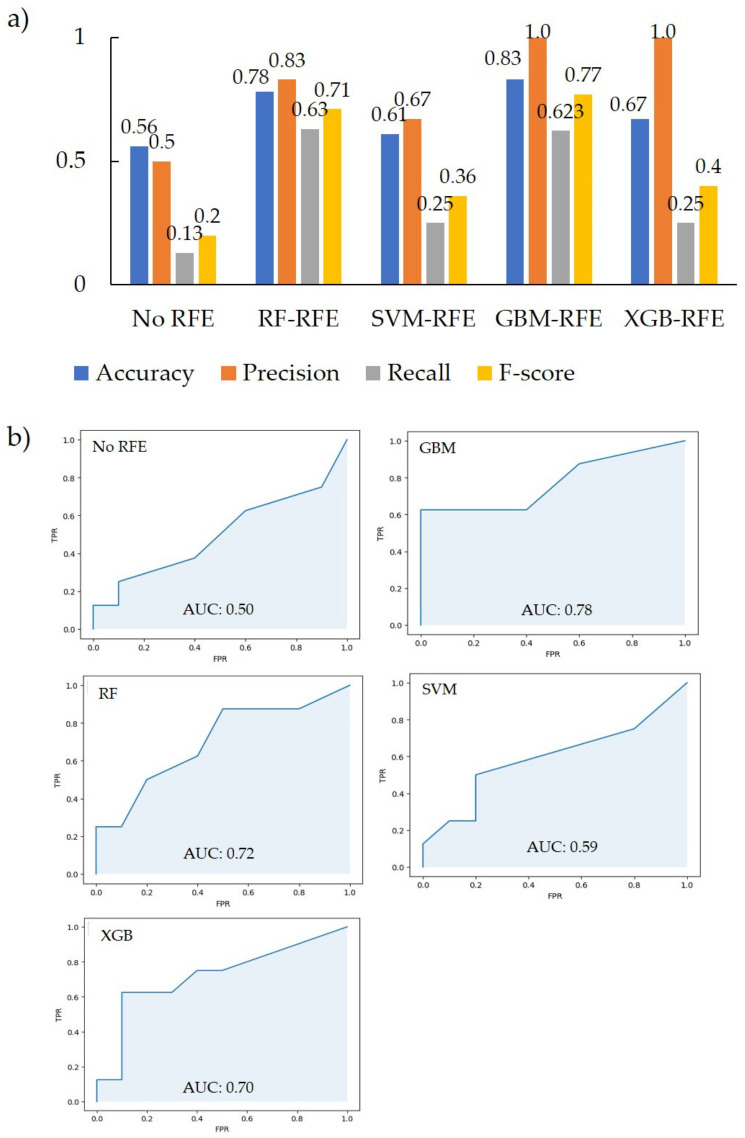
Performance of each model. (**a**) Accuracy, Precision, Recall, and F-score of each RFE; (**b**) ROC and AUC score of each RFE.

Feature selection was performed using the importance-based RFE method, and the importance was evaluated using the GBM, RF, SVM, and XGB classifiers. When a classifier model is trained on a training dataset, feature weights that reflect the importance of each feature are obtained. After all the features were ranked according to their weights, the feature with the lowest weight value was removed. The classifier is then retrained with the remaining features until there are no more features to learn. Finally, the model-based RFE method can obtain important features and show good performance [20]. The classification models were constructed by RF classifiers using the selected feature values above. As a result of feature reduction using GBM-RFE, RF-RFE, SVM-RFE, and XGB-RFE, the values of accuracy, precision, recall, and F-score for all models were higher than those obtained without RFE. The GBM-RFE showed the highest accuracy and F-score, the GBM-RFE and XGB-RFE had the highest precision, and the RF-RFE had the highest recall. From the results of the receiver operating characteristic curve and the area under the curve (AUC), GBM-RFE had the highest AUC value (Figure 4).

**Figure 5:**
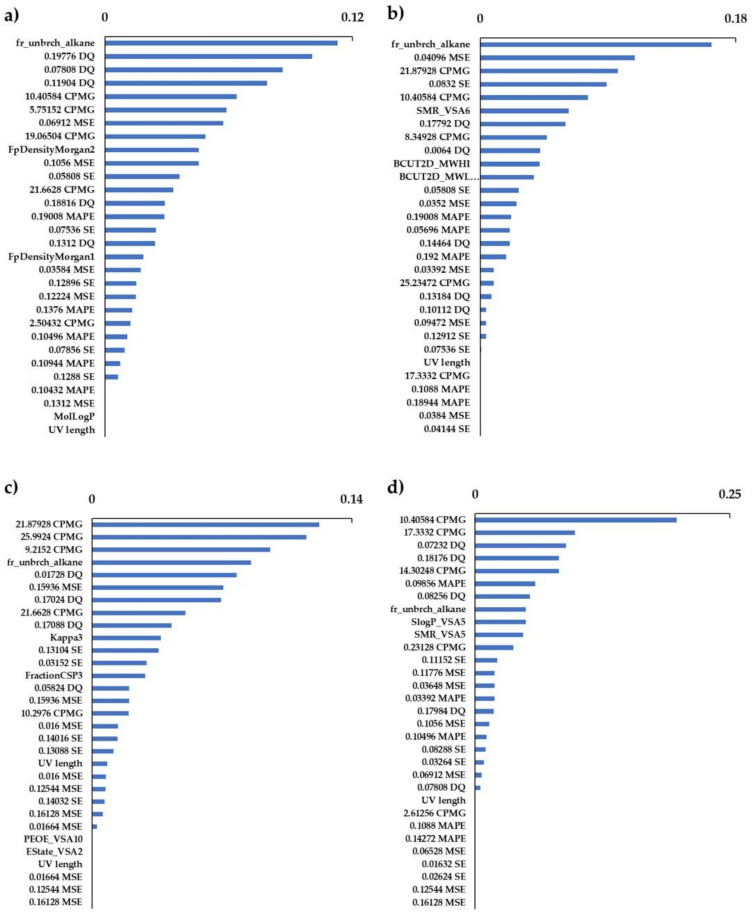
Important features of each RF classification model. Feature selections were performed by (**a**) GBM-RFE, (**b**) RF-RFE, (**c**) SVM-RFE, and (**d**) XGB-RFE.

**Figure 6:**
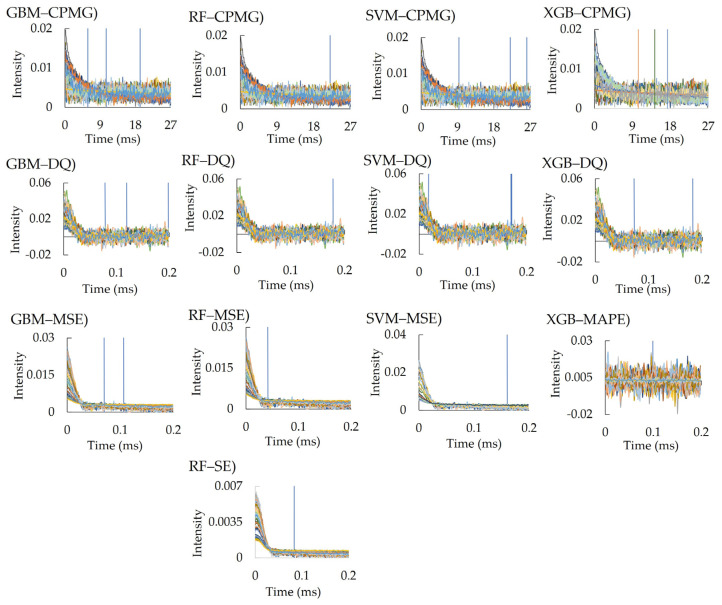
Important features of relaxation time in each pulse sequence. The extracted important features (blue line) for each relaxation time are shown.

The important factors extracted differed for each model (Figure 5). As a feature of RDKit, fr_unbrch_alkane was the top selected feature in all RFE models. For the TD-NMR sequence, several time points of the CPMG, double quantum (DQ) filter, MSE, MAPE, and SE sequences were selected, but most of them were after the intermediate region where the slope of the transition curve becomes gentle (Figure 6). The important factors extracted differed for each model (Figure 5 and Figure 6).

## 4. Discussion

Among the four RFE methods, GBM-RFE exhibited the best feature-selection performance. For LightGBM, following the decision tree analysis, gradient boosting was employed to enhance the accuracy. This boosting technique improves the predictive performance by learning from errors between predicted and actual values; it particularly focuses on data that could not be initially accurately predicted, and this method of growing according to the leaves of a decision tree is called leaf-wise [43].

Although XGB is based on gradient boosting, a difference with the branches of the decision tree, called level-wise, exists for each layer [43]. SVM-RFE, a wrapper method [44], was employed for feature selection in this study using a linear form [45]. However, the linear approach might not have effectively classified the current dataset, which comprised RDKit and five pulse sequence data with diverse characteristics. RF, a bagging method based on decision trees [46], demonstrated the second-best performance among the models. Its strength lies in amalgamating multiple decision trees into an ensemble, which potentially contributes to its effective analysis ability. Although both GBM and XGB are gradient boosting methods, their inherent approaches are different. GBM utilizes the leaf-wise method, focusing on improving accuracy while learning the errors for each leaf. Interestingly, GBM-RFE proved suitable for our dataset with its varying characteristics. Notably, the selection and importance of the features were dependent on the RFE methods employed in the analysis. This dependency underscores the significance of the chosen methodology in determining feature relevance and importance.

TD-NMR measurements offer a wide dynamic range, spanning from sub-microseconds (µs) to seconds, allowing for the extraction of various types of information across different time scales [46]. Mobility within meso-regions, like domain fluctuations, was observed in the µs range [40,47,48], while fluctuations in chain ends or the mobility of unfrozen and bound water were detected at the ms level [31]. The CPMG pulse sequence enables the measurement of long *T*_2_ relaxation times, allowing the analysis of the long components (liquid-like) of polymers [28,49,50,51]. DQ, MSE, and SE have short *T*_2_ relaxation times and are thus suitable for analysis of the rigid components (solid-like) of polymers [52,53,54]. The data after the middle region of each transitional curve includes data related to highly motile components. The monomers used in this study contained acrylamide groups, and the monomer FOM-03010 features a betaine structure, which is a zwitterionic group. The C=O and N=H of the acrylamide group [55] (betaine structures [56]) interact with the water surrounding the polymer, forming a hydration layer that inhibits foulant adhesion. In our case, multiple pulse sequences of CPMG, DQ, MSE, and SE were used to detect data from relaxation curves; in addition, various motilities might be involved in the measured contact angle and hydration properties.

Among the several molecular descriptors of physicochemical features, fr_unbrch_alkane (number of unbranched alkanes of at least four members, excluding halogenated alkanes) [57], which represented the proportion of unbranched alkanes, was selected in most of the RFE methods. The presence of branched alkanes in molecular structures is crucial for predicting crystallization owing to their disruptive effect on molecular packing, which destabilizes the liquid crystal phase [58]. This characteristic suggests that hydrophilic monomers may form structures conducive to expressing hydrophilicity by orderly bonding among themselves. Furthermore, molecular fluctuations occur more easily in a linear structure than in a branched structure. Based on the TD-NMR data, intermediate or late relaxation time and high molecular mobility were selected as important factors in the region; thus, we can infer that this molecular mobility (molecular fluctuation) is involved in the expression of hydrophilicity.

As discussed earlier, our current methodology offers the unique advantage of simultaneously providing the essential components from both molecular descriptors and TD-NMR *T_2_* relaxation curves. These components respectively represent physicochemical and dynamic properties. While understanding both properties is crucial for designing superior surface modifications, the challenge lies in the human capacity to manage a vast array of molecular descriptors and numerous NMR curves obtained through various pulse sequences. An additional noteworthy aspect is our utilization of diverse types of RFE methods. The importance attributed to ML algorithms varies, and relying on a single set of criteria can lead to misunderstandings due to factors like noise or pseudo-correlation. Therefore, our approach is particularly well-suited for a comprehensive and multifaceted examination of material data. In recent years, simple and inexpensive methods using smartphones and ML have been developed to measure contact angles [59,60]. Until recently, expensive contact angle meters were extensively used. However, these new ML-based methods enable the evaluation of the process of creating hydrophilic/hydrophobic polymer coating materials and aid in efficiently managing their manufacturing process. Therefore, incorporating the proposed ML-based method into future studies will be useful.

## 5. Conclusions

In our study, we showcased feature-selection techniques for molecular descriptors assessed via RDKit and relaxation curves obtained from TD-NMR, employing RFE for surface modifications. Our surface modifications involved copolymerizing various combinations of acrylamide monomers on PET films. To evaluate polymer chain dynamics across a broad time range, we utilized TD-NMR measurements with multiple pulse sequences, standardizing the data obtained from these five sequences. Applying GBM-RFE, RF-RFE, SCM-RFE, and XGB-RFE treatments to these descriptors significantly improved the predictability of the RF classifications. Moreover, our findings highlighted the crucial roles played by both the physicochemical properties and dynamics of polymer chains in determining the surface properties. The RFE method not only enhanced the predictability but also allowed us to extract critical factors or time regions from both physicochemical and TD-NMR data. This deeper insight into underlying mechanisms underscores the versatility of these approaches, extending their applicability beyond surface modification to other materials requiring comprehensive multi-modal information. 

## Data Availability

The data underlying this article will be shared on reasonable request to the corresponding author.

## Supplementary Materials

The following supporting information can be downloaded at: https://www.mdpi.com/article/10.3390/polym16060824/s1; Figure S1: chemical structure of monomers; Figure S2: The flow of data analysis of this study; Figure S3: Autoscaling of each *T_2_* relaxation curve; Figure S4: Performance of each criterion for binary classification; Table S1: list of samples; Table S2: list of the molecular descriptors; Table S3: list of the RDKit descriptors of each monomer; Table S4: the parameter of each random forest classifier model.

## References

[1] Vogelpohl T., Töller A.E. (2021). Perspectives on the bioeconomy as an emerging policy field. J. Environ. Policy Plan..

[2] Reike D., Vermeulen W.J.V., Witjes S. (2018). The circular economy: New or Refurbished as CE 3.0?—Exploring Controversies in the Conceptualization of the Circular Economy through a Focus on History and Resource Value Retention Options. Resour. Conserv. Recycl..

[3] Kakadellis S., Rosetto G. (2021). Achieving a circular bioeconomy for plastics. Science.

[4] Nasrabadi A.E., Ramavandi B., Bonyadi Z. (2023). Recent progress in biodegradation of microplastics by *Aspergillus* sp. in aquatic environments. Colloid. Interface Sci. Commun..

[5] Hossain S., Manan H., Shukri Z.N.A., Othman R., Kamaruzzan A.S., Rahim A.I.A., Khatoon H., Minhaz T.M., Islam Z., Kasan N.A. (2023). Microplastics biodegradation by biofloc-producing bacteria: An inventive biofloc technology approach. Microbiol. Res..

[6] Yoshida S., Hiraga K., Takehana T., Taniguchi I., Yamaji H., Maeda Y., Toyohara K., Miyamoto K., Kimura Y., Oda K. (2016). A bacterium that degrades and assimilates poly (ethylene terephthalate). Science.

[7] Vuong P., McKinley A., Kaur P. (2023). Understanding biofouling and contaminant accretion on submerged marine structures. NPJ Mater. Degrad..

[8] Qiu H., Feng K., Gapeeva A., Meurisch K., Kaps S., Li X., Yu L., Mishra Y.K., Adelung R., Baum M. (2022). Functional polymer materials for modern marine biofouling control. Prog. Polymr Sci..

[9] Kadoma Y., Nakabayashi N., Masuhara E., Yamauchi J. (1978). Synthesis and Hemolysis Test of the Polymer Containing Phosphorylcholine Groups. Koubunshi Ronbunshu.

[10] Ishihara K., Nomura H., Mihara T., Kurita K., Iwasaki Y., Nakabayashi N. (1998). Why do phospholipid polymers reduce protein adsorption?. J. Biomed. Mater. Res..

[11] Tanaka M., Motomura T., Kawada M., Anzai T., Kasori Y., Shiroya T., Shimura K., Onishi M., Mochizuki A. (2000). Blood compatible aspects of poly (2-methoxyethylacrylate) (PMEA)—Relationship between protein adsorption and platelet adhesion on PMEA surface. Biomaterials.

[12] Sato K., Kobayashi S., Kusakari M., Watahiki S., Oikawa M., Hoshiba T., Tanaka M. (2015). The Relationship Between Water Structure and Blood Compatibility in Poly (2-methoxyethyl Acrylate) (PMEA) Analogues. Macromol. Biosci..

[13] Ashok D., Cheeseman S., Wang Y., Funnell B., Leung S.F., Tricoli A., Nisbet D. (2023). Superhydrophobic surfaces to combat bacterial surface colonization. Adv. Mater. Interfaces.

[14] Hu P., Xie Q., Ma C., Zhang G. (2020). Silicone-based fouling-release coatings for marine antifouling. Langmuir.

[15] Webber M.J., Tibbitt M.W. (2022). Dynamic and reconfigurable materials from reversible network interactions. Nat. Rev. Mater..

[16] Carré A., Gastel J.-C., Shanahan M.E.R. (1996). Viscoelastic effects in the spreading of liquids. Nature.

[17] Matlahov I., van der Wel P.C. (2018). Hidden motions and motion-induced invisibility: Dynamics-based spectral editing in solid-state NMR. Methods.

[18] Gupta S., Puttaiahgowda Y.M., Parambil A.M., Kulal A. (2023). Fabrication of crosslinked piperazine polymer coating: Synthesis, characterization and its activity towards microorganisms. J. Mol. Struct..

[19] Tsuji T., Ono T., Taguchi H., Leong K.H., Hayashi Y., Kumada S., Okada K., Onuki Y. (2023). Continuous Monitoring of the Hydration Behavior of Hydrophilic Matrix Tablets Using Time-Domain NMR. Chem. Pharm. Bull..

[20] Colnago L.A., Wiesman Z., Pages G., Musse M., Monaretto T., Windt C.W., Rondeau-Mouro C. (2021). Low field, time domain NMR in the agriculture and agrifood sectors: An overview of applications in plants, foods and biofuels. J. Magn. Reson..

[21] Matsumura T., Nagamura N., Akaho S., Nagata K., Ando Y. (2019). Spectrum adapted expectation-maximization algorithm for high-throughput peak shift analysis. Sci. Technol. Adv. Mater..

[22] Bolón-Canedo V., Sánchez-Maroño N., Alonso-Betanzos A. (2016). Feature selection for high-dimensional data. Prog. Artif. Intell..

[23] Kusaka Y., Hasegawa T., Kaji H. (2019). Noise reduction in solid-state NMR spectra using principal component analysis. J. Phys. Chem. A.

[24] Peng W.K., Ng T.-T., Loh T.P. (2020). Machine learning assistive rapid, label-free molecular phenotyping of blood with two-dimensional NMR correlational spectroscopy. Commun. Biol..

[25] Florentino-Ramos E., Villa-Ruano N., Hidalgo-Martínez D., Ramírez-Meraz M., Méndez-Aguilar R., Velásquez-Valle R., Zepeda-Vallejo L.G., Pérez-Hernández N., Becerra-Martínez E. (2019). ^1^H NMR-based fingerprinting of eleven Mexican Capsicum annuum cultivars. Food Res. Int..

[26] Zhou T., Song Z., Sundmacher K. (2019). Big data creates new opportunities for materials research: A review on methods and applications of machine learning for materials design. Engineering.

[27] Olfatbakhsh T., Andrews J.L., Milani A.S. (2022). Materials informatics of woven fabric composites: Effect of different dimensionality reduction and learning methods. Mater. Today Commun..

[28] Yamada S., Tsuboi Y., Yokoyama D., Kikuchi J. (2023). Polymer composition optimization approach based on feature extraction of bound and free water using time-domain nuclear magnetic resonance. J. Magn. Reson..

[29] Forshed J., Schuppe-Koistinen I., Jacobsson S.P. (2003). Peak alignment of NMR signals by means of a genetic algorithm. Anal. Chim. Acta.

[30] Cho H.-W., Kim S.B., Jeong M.K., Park Y., Ziegler T.R., Jones D.P. (2008). Genetic algorithm-based feature selection in high-resolution NMR spectra. Expert. Syst. Appl..

[31] Wei F., Fukuchi M., Ito K., Sakata K., Asakura T., Date Y., Kikuchi J. (2020). Large-scale evaluation of major soluble macromolecular components of fish muscle from a conventional ^1^H-NMR spectral database. Molecules.

[32] Munir N., McMorrow R., Mulrennan K., Whitaker D., McLoone S., Kellomäki M., Talvitie E., Lyyra I., McAfee M. (2023). Interpretable Machine Learning Methods for Monitoring Polymer Degradation in Extrusion of Polylactic Acid. Polymers.

[33] Fallah Atanaki F., Behrouzi S., Ariaeenejad S., Boroomand A., Kavousi K. (2020). BIPEP: Sequence-based prediction of biofilm inhibitory peptides using a combination of nmr and physicochemical descriptors. ACS Omega.

[34] Jeon H., Oh S. (2020). Hybrid-recursive feature elimination for efficient feature selection. Appl. Sci..

[35] Zhang H., Zhu S., Yang J., Ma A. (2022). Advancing strategies of biofouling control in water-treated polymeric membranes. Polymers.

[36] Morita S., Tanaka M., Ozaki Y. (2007). Time-resolved in situ ATR-IR observations of the process of sorption of water into a poly (2-methoxyethyl acrylate) film. Langmuir.

[37] Noto N., Yada A., Yanai T., Saito S. (2023). Machine-Learning Classification for the Prediction of Catalytic Activity of Organic Photosensitizers in the Nickel (II)-Salt-Induced Synthesis of Phenols. Angew. Chem. Int. Ed..

[38] Feng Z., Cheng Y., Khlyustova A., Wani A., Franklin T., Varner J.D., Hook A.L., Yang R. (2023). Virtual High-Throughput Screening of Vapor-Deposited Amphiphilic Polymers for Inhibiting Biofilm Formation. Adv. Mater. Technol..

[39] Ono S., Hewage H.T., Visvanathan C. (2023). Towards Plastic Circularity: Current Practices in Plastic Waste Management in Japan and Sri Lanka. Sustainability.

[40] Schäler K., Roos M., Micke P., Golitsyn Y., Seidlitz A., Thurn-Albrecht T., Schneider H., Hempel G., Saalwächter K. (2015). Basic principles of static proton low-resolution spin diffusion NMR in nanophase-separated materials with mobility contrast. Solid. State Nucl. Magn. Reson..

[41] Takamura A., Tsukamoto K., Sakata K., Kikuchi J. (2021). Integrative measurement analysis via machine learning descriptor selection for investigating physical properties of biopolymers in hairs. Sci. Rep..

[42] Weininger D. (1988). SMILES, a chemical language and information system. 1. Introduction to methodology and encoding rules. J. Chem. Inf. Comput. Sci..

[43] Suenaga D., Takase Y., Abe T., Orita G., Ando S. (2023). Prediction accuracy of Random Forest, XGBoost, LightGBM, and artificial neural network for shear resistance of post-installed anchors. Structures.

[44] Duan K.-B., Rajapakse J.C., Wang H., Azuaje F. (2005). Multiple SVM-RFE for gene selection in cancer classification with expression data. IEEE Trans. Nanobiosci..

[45] Ding Y., Wilkins D. (2006). Improving the performance of SVM-RFE to select genes in microarray data. BMC Bioinform..

[46] Altman N., Krzywinski M. (2017). Ensemble methods: Bagging and random forests. Nat. Meth..

[47] Schlagnitweit J., Tang M., Baias M., Richardson S., Schantz S., Emsley L. (2015). A solid-state NMR method to determine domain sizes in multi-component polymer formulations. J. Magn. Reson..

[48] Hara K., Yamada S., Kurotani A., Chikayama E., Kikuchi J. (2022). Materials informatics approach using domain modelling for exploring structure-property relationships of polymers. Sci. Rep..

[49] Borgia G., Fantazzini P., Ferrando A., Maddinelli G. (2001). Characterisation of crosslinked elastomeric materials by ^1^H NMR relaxation time distributions. Magn. Reson. Imag..

[50] Dare D., Chadwick D. (1996). A low resolution pulsed nuclear magnetic resonance study of epoxy resin during cure. Int. J. Adhes. Adhes..

[51] Li J., Ma E. (2021). Characterization of water in wood by time-domain nuclear magnetic resonance spectroscopy (TD-NMR): A review. Forests.

[52] Grunin L., Ivanova M., Schiraya V., Grunina T. (2023). Time-Domain NMR Techniques in Cellulose Structure Analysis. Appl. Magn. Reson..

[53] Garcia R.H.d.S., Filgueiras J.G., Colnago L.A., de Azevedo E.R. (2022). Real-Time Monitoring Polymerization Reactions Using Dipolar Echoes in 1H Time Domain NMR at a Low Magnetic Field. Molecules.

[54] Uguz S.S., Ozel B., Grunin L., Ozvural E.B., Oztop M.H. (2022). Non-Conventional Time Domain (TD)-NMR Approaches for Food Quality: Case of Gelatin-Based Candies as a Model Food. Molecules.

[55] Futscher M.H., Philipp M., Müller-Buschbaum P., Schulte A. (2017). The Role of Backbone Hydration of Poly(N-isopropyl acrylamide) Across the Volume Phase Transition Compared to its Monomer. Sci. Rep..

[56] Ishihara K., Mu M., Konno T., Inoue Y., Fukazawa K. (2017). The unique hydration state of poly(2-methacryloyloxyethyl phosphorylcholine). J. Biomater. Sci. Polym. Ed..

[57] Shamsara J. (2019). A random forest model to predict the activity of a large set of soluble epoxide hydrolase inhibitors solely based on a set of simple fragmental descriptors. Comb. Chem. High. Throughput Screen..

[58] Chen C.-H., Tanaka K., Kotera M., Funatsu K. (2020). Comparison and improvement of the predictability and interpretability with ensemble learning models in QSPR applications. J. Cheminf..

[59] Kabir H., Garg N. (2023). Machine learning enabled orthogonal camera goniometry for accurate and robust contact angle measurements. Sci. Rep..

[60] Chen H., Muros-Cobos J.L., Amirfazli A. (2018). Contact angle measurement with a smartphone. Rev. Sci. Instrum..

