# Supplementary material for: Designing Sustainable Hydrophilic Interfaces via Feature Selection from Molecular Descriptors and Time-Domain Nuclear Magnetic Resonance Relaxation Curves
Source: Polymers (Basel). 2024 Mar 15;16(6):824. doi: 10.3390/polym16060824 (PMC10975876; doi:10.3390/polym16060824)
Supplement: Supplementary file 1 [file polymers-16-00824-s001.zip › polymers-2875018-supplementary.pdf]

Supplementary materials

Designing Sustainable Hydrophilic Interfaces via Feature Selection from Molecular Descriptors and Time-Domain Nuclear Magnetic Resonance Relaxation Curves

Masayuki Okada [1, 3], Yoshifumi Amamoto [2, 3] and Jun Kikuchi [1, 3, 4,*]

1 Graduate School of Bioagricultural Sciences, Nagoya University, 1 Furo–cho, Chikusa–ku, Nagoya 464–8601, Japan; masayuki.okada@a.riken.jp
2 Graduate School of Social Data Science, Hitotsubashi University, 2–1 Naka, Kunitachi 186–8601, Japan; yoshifumi.amamoto@a.riken.jp
3 RIKEN Center for Sustainable Resource Science, 1–7–22 Suehiro–cho, Tsurumi–ku, Yokohama 230–0045, Japan
4 Graduate School of Medical Life Science, Yokohama City University, 1-7-29 Suehiro-cho, Tsurumi-ku, Yokohama, 230-0045, Japan

* Correspondence: jun.kikuchi@riken.jp; Tel.: +81–45–508–7221

Content

Supplementary materials 1
Figure S1. Chemical structure of monomers. 2
Figure S2. The flow of data analysis of this study. 3
Figure S3. Autoscaling of each T_2 relaxation curve. 4
Figure S4. Performance of each criterion for binary classification. 5
Table S1. List of samples. 6
Table S2. List of the molecular descriptors. 8
Table S3. List of the RDKit descriptors of each monomer. 9
Table S4. The parameter of each Random Forest classifier model. 12

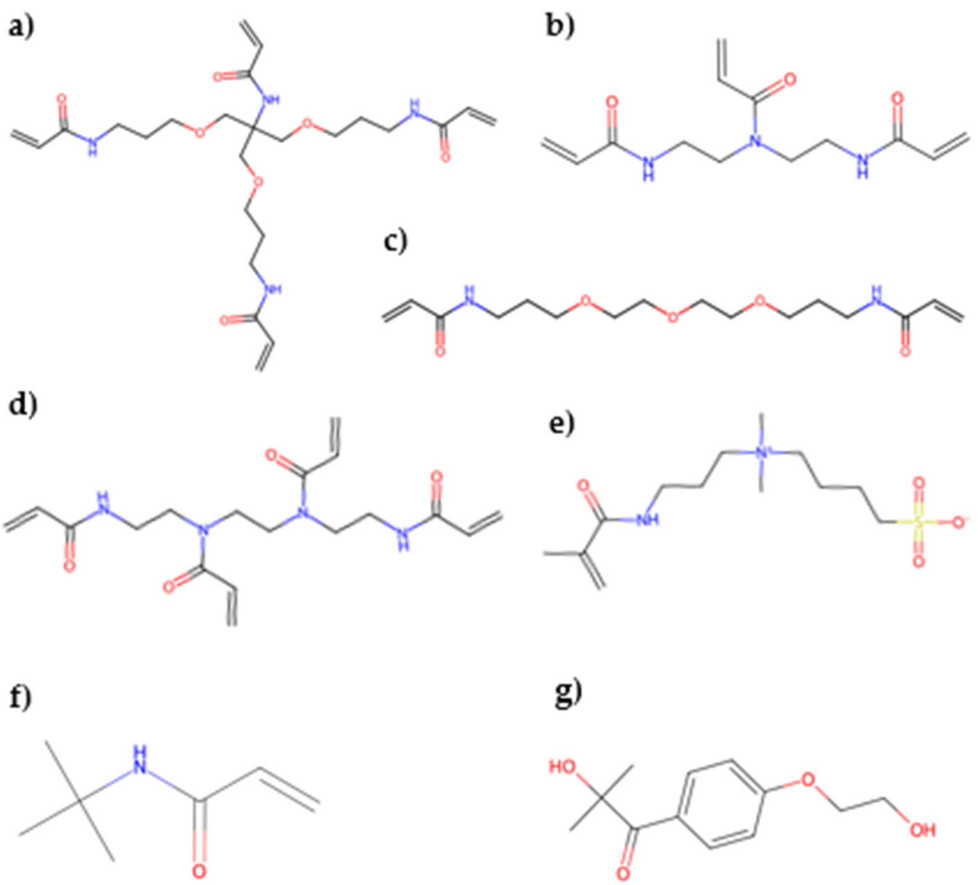

Figure S1. Chemical structure of monomers.

This figure illustrates the chemical structures. a) FOM-3006）, b) FOM-3007, c) FOM-3008, d) FOM-3009）, e) FOM-3010）, f) NTBA, g) Irgacure 2959.

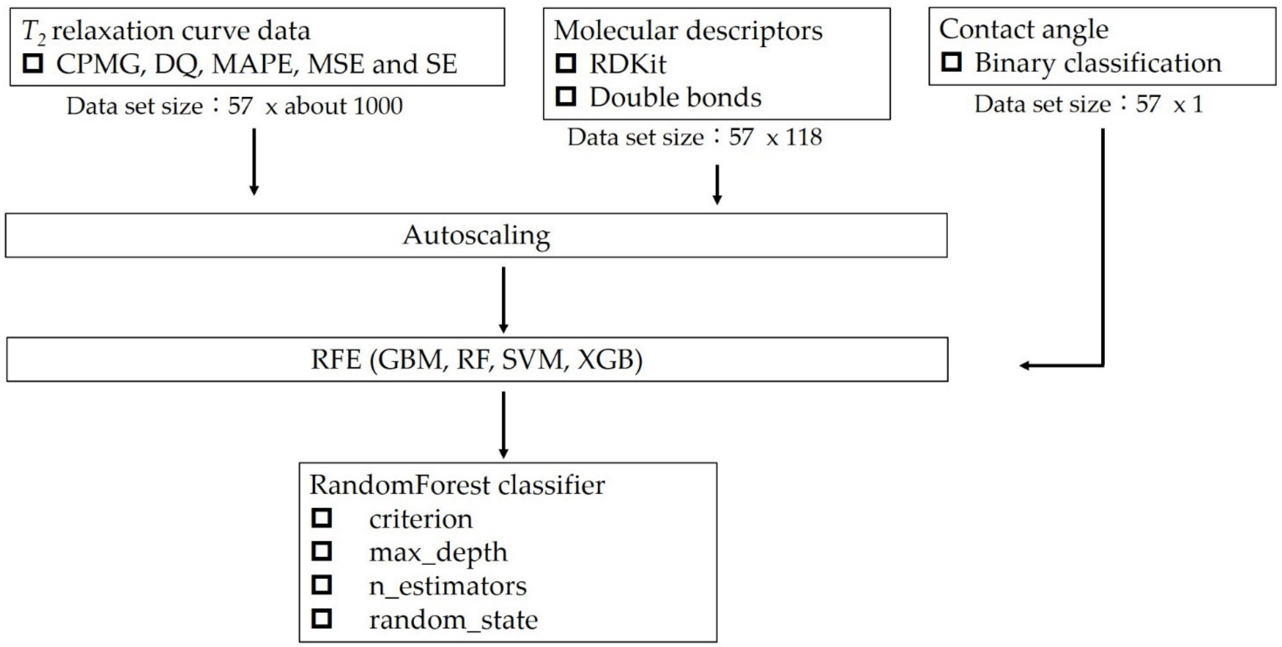

Figure S2. The flow of data analysis of this study.

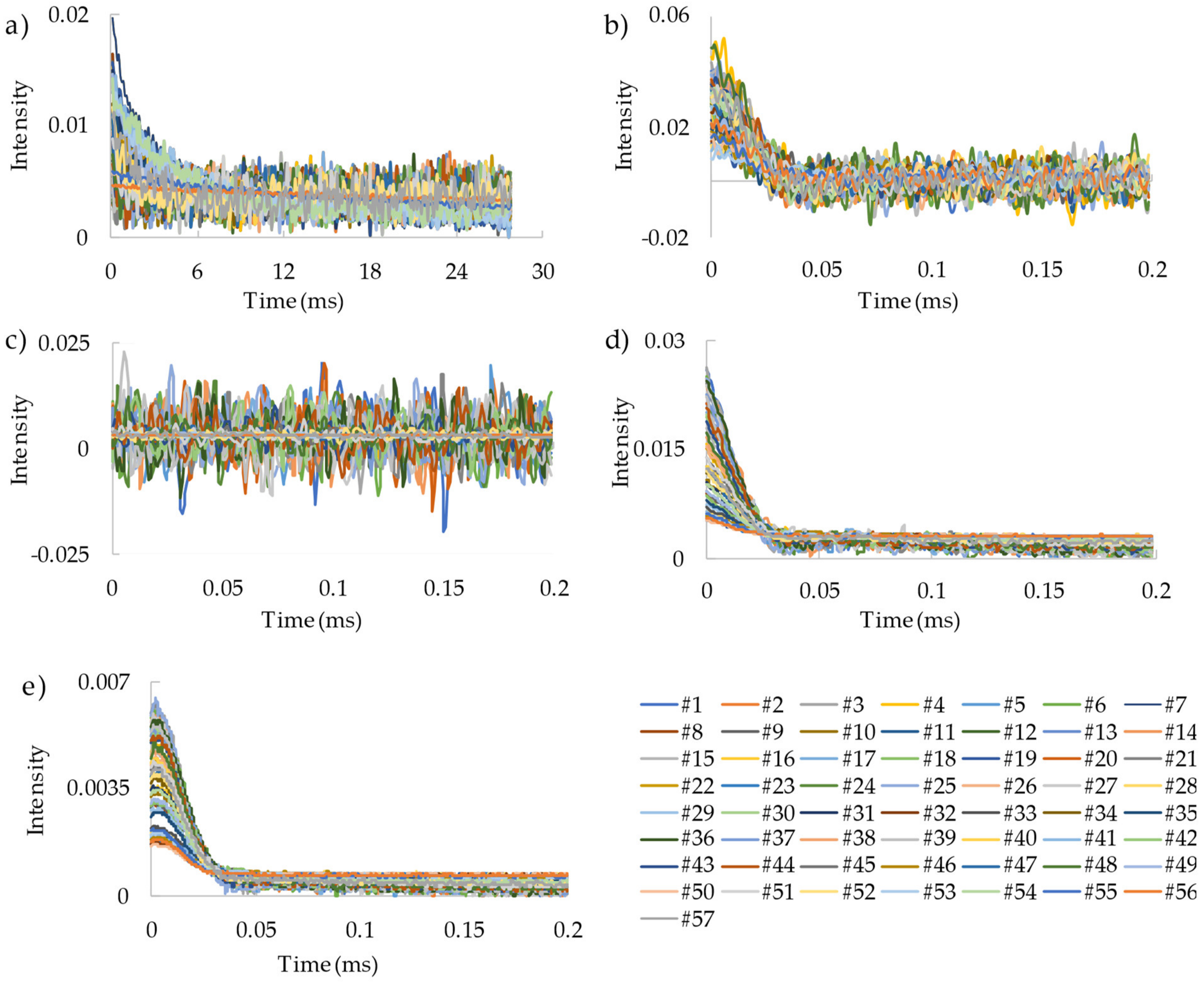

Figure S3. Autoscaling of each T_2 relaxation curve.

This figure illustrates autoscaling of each sample. a) CPMG, b) DQ, c) MAPE, d) MSE, e) SE

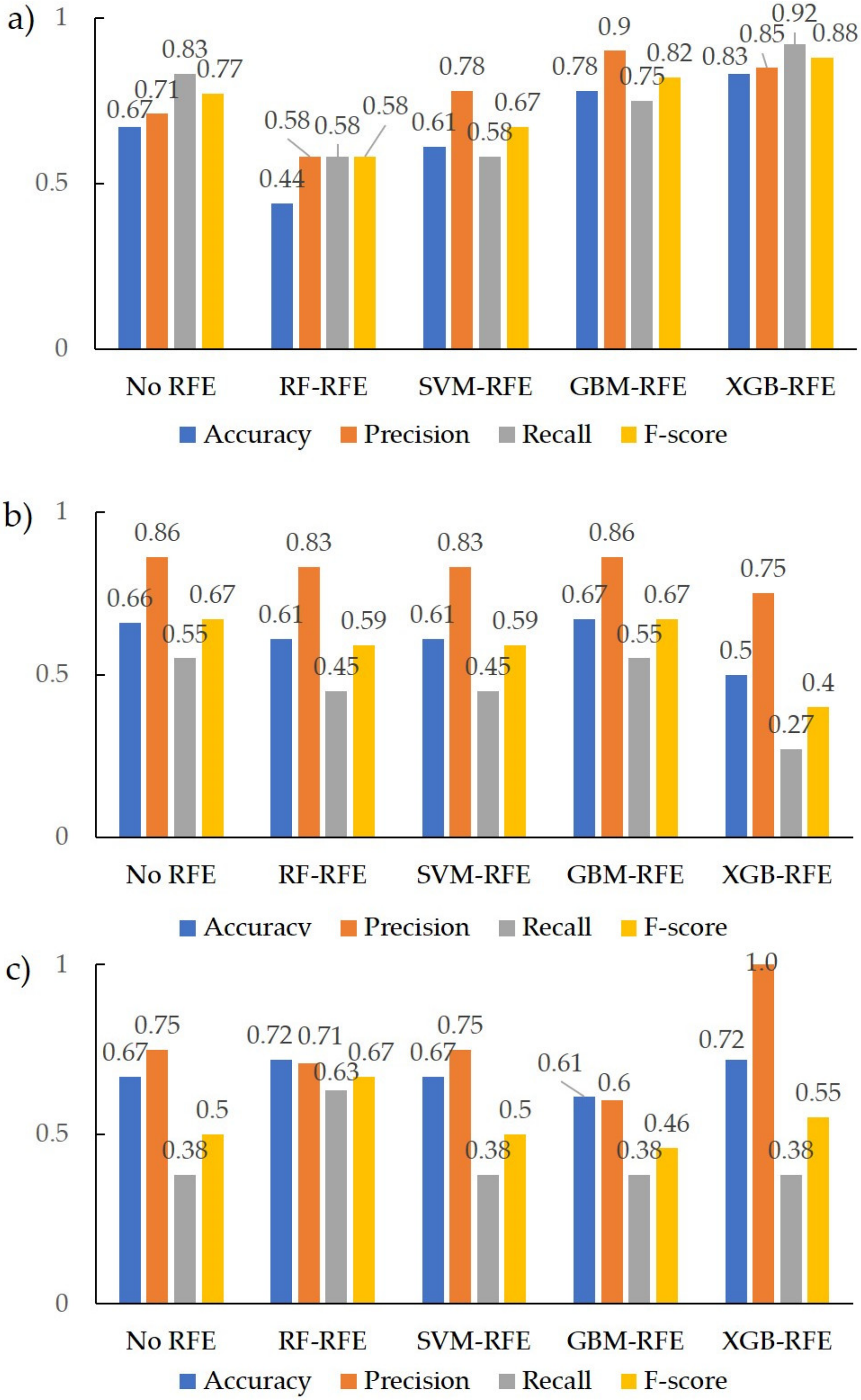

Figure S4. Performance of each criterion for binary classification. a) 25°, b) 30°, c) 35°

Table S1. List of samples.
This table demonstrates the composition of each polymer and process data including dry temperature, dry time, UV length and IV irradiation amount.

No.	FOM-03006(wt%)	FOM-03007(wt%)	FOM-03008(wt%)	FOM-03009(wt%)	FOM-03010(wt%)	NTBA(wt%)	Irgacure 2959(wt%)	Dry temperature (°C)	Dry time (min)	UV length (nm)	UV Irradiation Amount (J/cm^2)
1	48.5	48.5	0	0	0	0	3	50	10	365	3
2	48.5	0	48.5	0	0	0	3	50	10	365	3
3	48.5	0	0	48.5	0	0	3	50	10	365	3
4	0	48.5	0	0	48.5	0	3	50	10	365	3
5	0	48.5	48.5	0	0	0	3	50	10	365	3
6	0	0	0	97	0	0	3	50	10	365	3
7	48.5	0	0	0	0	48.5	3	50	10	365	3
8	0	48.5	0	0	0	48.5	3	50	10	365	3
9	0	0	48.5	0	0	48.5	3	50	10	365	3
10	0	0	0	48.5	0	48.5	3	50	10	365	3
11	0	0	0	0	48.5	48.5	3	50	10	365	3
12	0	48.5	48.5	0	0	0	3	50	10	365	3
13	0	0	48.5	48.5	0	0	3	50	10	365	3
14	0	48.5	0	48.5	0	0	3	50	10	365	3
15	48.5	48.5	0	0	0	0	3	50	10	365	3
16	48.5	0	48.5	0	0	0	3	50	10	365	3
17	48.5	0	0	48.5	0	0	3	50	10	365	3
18	48.5	0	0	0	48.5	0	3	50	10	365	3
19	48.5	0	0	0	0	48.5	3	50	10	365	3
20	0	48.5	48.5	0	0	0	3	50	10	365	3
21	0	48.5	0	48.5	0	0	3	50	10	365	3
22	0	48.5	0	0	48.5	0	3	50	10	365	3
23	0	48.5	0	0	0	48.5	3	50	10	365	3
24	97	0	0	0	0	0	3	50	10	365	3
25	0	97	0	0	0	0	3	50	10	365	3
26	0	0	97	0	0	0	3	50	10	365	3
27	0	0	0	97	0	0	3	50	10	365	3
28	0	0	0	0	0	0	3	50	10	365	3
29	0	0	0	0	0	97	3	50	10	365	3
30	0	0	0	0	0	0	3	50	10	365	3
31	0	0	48.5	0	48.5	0	3	50	10	365	3
32	0	0	48.5	0	0	48.5	3	50	10	365	3
33	0	0	0	0	48.5	48.5	3	50	10	365	3
34	0	0	0	48.5	48.5	0	3	50	10	365	3
35	0	0	0	0	48.5	48.5	3	50	10	365	3

36	97	0	0	0	0	0	3	50	10	365	3
37	48.5	48.5	0	0	0	0	3	50	10	365	3
38	48.5	0	48.5	0	0	0	3	50	10	365	3
39	48.5	0	0	48.5	0	0	3	50	10	365	3
40	48.5	0	0	0	0	0	3	50	10	365	3
41	48.5	0	0	0	0	48.5	3	50	10	365	3
42	0	97	0	0	0	0	3	50	10	365	3
43	0	48.5	48.5	0	0	0	3	50	10	365	3
44	0	48.5	0	48.5	0	0	3	50	10	365	3
45	0	48.5	0	0	48.5	0	3	50	10	365	3
46	0	48.5	0	0	0	48.5	3	50	10	365	3
47	0	0	97	0	0	0	3	50	10	365	3
48	0	0	48.5	48.5	0	0	3	50	10	365	3
49	0	0	48.5	0	48.5	0	3	50	10	365	3
50	0	0	48.5	0	0	48.5	3	50	10	365	3
51	0	0	0	97	0	0	3	50	10	365	3
52	0	0	0	48.5	48.5	0	3	50	10	365	3
53	0	0	0	48.5	0	48.5	3	50	10	365	3
54	0	0	0	0	97	0	3	50	10	365	3
55	0	0	0	0	48.5	0	3	50	10	365	3
56	0	0	0	0	0	97	3	50	10	365	3
57	48.5	0	0	0	48.5	0	3	50	10	365	3

Table S2. List of the molecular descriptors.
This table demonstrates the molecular descriptors including 114 RDKit descriptors and 2 descriptors related to double bonds (double bonds number, double bonds length) used in this study.

1	Double bonds number	31	Chi0n	61	SMR_VSA5	91	FractionCSP3
2	Double bonds length	32	Chi0v	62	SMR_VSA6	92	HeavyAtomCount
3	MaxAbsEStateIndex	33	Chi1	63	SMR_VSA7	93	NHOHCount
4	MaxEStateIndex	34	Chi1n	64	SMR_VSA9	94	NOCount
5	MinAbsEStateIndex	35	Chi1v	65	SlogP_VSA1	95	NumAromaticCarbocycles
6	MinEStateIndex	36	Chi2n	66	SlogP_VSA11	96	NumAromaticRings
7	Qed	37	Chi2v	67	SlogP_VSA2	97	NumHAcceptors
8	MolWt	38	Chi3n	68	SlogP_VSA3	98	NumHDonors
9	HeavyAtomMolWt	39	Chi3v	69	SlogP_VSA5	99	NumHeteroatoms
10	ExactMolWt	40	Chi4n	70	SlogP_VSA6	100	NumRotatableBonds
11	NumValenceElectrons	41	Chi4v	71	TPSA	101	RingCount
12	MaxPartialCharge	42	HallKierAlpha	72	EState_VSA1	102	MolLogP
13	MinPartialCharge	43	Ipc	73	EState_VSA10	103	MolMR
14	MaxAbsPartialCharge	44	Kappa1	74	EState_VSA2	104	fr_Al_OH
15	MinAbsPartialCharge	45	Kappa2	75	EState_VSA3	105	fr_Al_OH_noTert
16	FpDensityMorgan1	46	Kappa3	76	EState_VSA4	106	fr_C_O
17	FpDensityMorgan2	47	LabuteASA	77	EState_VSA5	107	fr_C_O_noCOO
18	FpDensityMorgan3	48	PEOE_VSA1	78	EState_VSA6	108	fr_NH0
19	BCUT2D_MWHI	49	PEOE_VSA10	79	EState_VSA7	109	fr_NH1
20	BCUT2D_MWLOW	50	PEOE_VSA11	80	EState_VSA8	110	fr_amide
21	BCUT2D_CHGHI	51	PEOE_VSA12	81	EState_VSA9	111	fr_benzene
22	BCUT2D_CHGLO	52	PEOE_VSA2	82	VSA_EState1	112	fr_ether
23	BCUT2D_LOGPHI	53	PEOE_VSA3	83	VSA_EState2	113	fr_ketone
24	BCUT2D_LOGPLOW	54	PEOE_VSA6	84	VSA_EState3	114	fr_ketone_Topliss
25	BCUT2D_MRHI	55	PEOE_VSA7	85	VSA_EState4	115	fr_quatN
26	BCUT2D_MRLOW	56	PEOE_VSA8	86	VSA_EState5	116	fr_unbrch_alkane
27	AvgIpc	57	PEOE_VSA9	87	VSA_EState6		
28	BalabanJ	58	SMR_VSA1	88	VSA_EState7		
29	BertzCT	59	SMR_VSA10	89	VSA_EState8		
30	Chi0	60	SMR_VSA3	90	VSA_EState9		

Table S3. List of the RDKit descriptors of each monomer.
This table demonstrates the 115 RDKit descriptors of 7 monomers used in this study.

Monomer	Double bonds number	Double bonds length	MaxAbsEStateIndex	MaxEState Index	MinAbsEState Index	MinEState Index	Qed	MolWt	HeavyAtom MolWt	ExactMolWt
FOM-3006	31	45.4	12.19059	12.19059	0.085699	-1.00956	0.113633	508.616	468.296	508.2897
FOM-3007	15	21.73	11.54465	11.54465	0.263237	-0.30004	0.557068	265.313	246.161	265.1426
FOM-3008	20	29.3	10.84992	10.84992	0.169982	-0.16998	0.316768	328.409	300.185	328.1998
FOM-3009	21	30.44	11.93471	11.93471	0.239879	-0.33572	0.436435	362.43	336.222	362.1954
FOM-3010	1	1.34	11.26999	11.26999	0.128742	-4.09314	0.275038	306.428	280.22	306.1613
NTBA	1	1.34	10.59826	10.59826	0.122685	-0.14755	0.524698	127.187	114.083	127.0997
Irgacure2959	0	0	11.66073	11.66073	0.051237	-1.36759	0.732459	224.256	208.128	224.1049

Monomer	NumValence Electrons	MaxPartial Charge	MinPartial Charge	MaxAbsPartialCharge	MinAbsPartial Charge	FpDensity Morgan1	FpDensity Morgan2	FpDensity Morgan3	BCUT2D_ MWHI	BCUT2D_ MWLOW
FOM-3006	202	0.24355	-0.37876	0.37876	0.24355	0.555556	0.861111	1.138889	16.50681	10.02779
FOM-3007	104	0.245578	-0.35079	0.350793	0.245578	0.842105	1.263158	1.578947	16.15549	10.36727
FOM-3008	132	0.242883	-0.37899	0.37899	0.242883	0.695652	1.043478	1.391304	16.54098	10.4124
FOM-3009	142	0.245578	-0.35079	0.350793	0.245578	0.615385	0.961538	1.230769	16.15847	10.32465
FOM-3010	118	0.24582	-0.74794	0.747944	0.24582	1.2	1.75	2.25	32.23974	10.23442
NTBA	52	0.24328	-0.34798	0.347976	0.24328	1.555556	2	2.111111	16.14887	10.10978
Irgacure2959	88	0.193254	-0.49121	0.491206	0.193254	1.3125	1.875	2.3125	16.48658	9.968691

Monomer	BCUT2D_ CHGHI	BCUT2D_ CHGLO	BCUT2D_ LOGPHI	BCUT2D_ LOGPLOW	BCUT2D_ MRHI	BCUT2D_ MRLOW	AvgIpc	BalabanJ	BertzCT	Chi0
FOM-3006	2.327595	-2.33653	2.102823	-2.58058	5.877063	-0.12313	2.886355	4.928565	661.0863	27.07286
FOM-3007	2.095136	-2.20825	1.896139	-2.41188	5.881347	-0.12655	2.4351	4.157077	351.3297	14.67336
FOM-3008	1.951516	-2.07086	1.855952	-2.22876	5.865812	-0.11688	2.57105	3.076284	315.3623	17.17552
FOM-3009	2.150445	-2.26581	1.962842	-2.44555	5.887123	-0.12966	2.663654	4.702344	521.5384	19.94938
FOM-3010	2.139607	-2.29616	2.040424	-2.42239	7.851944	-0.89017	2.285637	3.791167	429.6521	15.81155
NTBA	2.095136	-2.18159	1.966061	-2.38938	5.868368	-0.11731	1.615959	3.736394	121.3681	7.491564
Irgacure2959	2.243487	-2.15089	2.163889	-2.30477	6.012735	0.048766	2.246391	2.750872	348.2767	12.1818

Monomer	Chi0n	Chi0v	Chi1	Chi1n	Chi1v	Chi2n	Chi2v	Chi3n	Chi3v	Chi4n
FOM-3006	20.98085	20.98085	17.31803	11.70756	11.70756	7.677578	7.677578	4.55906	4.55906	2.722206
FOM-3007	10.85376	10.85376	9.044443	5.766312	5.766312	3.519713	3.519713	2.218988	2.218988	1.233747
FOM-3008	13.68122	13.68122	11.20191	7.741253	7.741253	4.534527	4.534527	2.644173	2.644173	1.457864
FOM-3009	14.90789	14.90789	12.38698	8.023422	8.023422	5.049123	5.049123	3.345117	3.345117	1.936458
FOM-3010	12.64531	13.46181	9.034293	6.726789	8.304139	5.741638	7.265241	3.021173	3.809848	1.790373
NTBA	6.192705	6.192705	3.954507	2.901048	2.901048	2.943375	2.943375	0.683595	0.683595	0.42063
Irgacure2959	9.434538	9.434538	7.447707	5.058125	5.058125	3.984587	3.984587	2.03219	2.03219	1.219255

Monomer	Chi4v	HallKier Alpha	Ipc	Kappa1	Kappa2	Kappa3	LabuteASA	PEOE_VSA1	PEOE_VSA10	PEOE_VSA11
FOM-3006	2.722206	-3.28	38291379	32.72	21.03935	17.19889	212.9256	35.47774	5.538925	0
FOM-3007	1.233747	-2.37	11705.53	16.63	9.638777	7.535466	112.3939	15.53349	0	0

FOM-3008	1.457864	-1.7	98684.6	21.3	16.66677	16.49704	137.5976	24.84417	0	0
FOM-3009	1.936458	-3.16	320152.6	22.84	13.16688	9.212625	153.4503	20.4334	0	0
FOM-3010	2.34805	-0.92	9766.525	19.08	8.385309	10.95299	121.7388	14.35257	0	0
NTBA	0.42063	-0.79	53.32665	8.21	2.66727	5.0749	55.95068	5.316789	0	0
Irgacure2959	1.219255	-1.39	2974.222	12.67845	5.094861	3.324899	94.48459	14.94992	17.95744	5.783245

Monomer	PEOE_VSA12	PEOE_VSA2	PEOE_VSA3	PEOE_VSA6	PEOE_VSA7	PEOE_VSA 8	PEOE_VSA9	SMR_VSA1	SMR_VSA10	SMR_VSA3
FOM-3006	23.62872	19.17815	0	26.31574	43.56655	39.45492	19.82065	33.38874	23.62872	21.26715
FOM-3007	17.72154	14.38361	0	19.73681	18.22806	26.17903	0	14.38361	17.72154	15.53349
FOM-3008	11.81436	9.589074	0	13.15787	24.99368	26.30328	26.42753	23.79966	11.81436	10.63358
FOM-3009	23.62872	19.17815	0	26.31574	24.30408	39.26854	0	19.17815	23.62872	20.4334
FOM-3010	5.90718	4.794537	8.417797	6.578936	19.76538	24.29154	37.30298	22.24811	16.02531	5.316789
NTBA	5.90718	4.794537	0	6.578936	26.84723	5.538925	0	4.794537	5.90718	5.316789
Irgacure2959	0	4.794537	0	0	38.11294	5.563451	6.606882	19.74445	5.783245	0

Monomer	SMR_VSA5	SMR_VSA6	SMR_VSA7	SMR_VSA9	SlogP_VSA1	SlogP_VSA1 1	SlogP_VSA2	SlogP_VSA3	SlogP_VSA5	SlogP_VSA6
FOM-3006	24.80139	59.27556	50.61982	0	21.26715	0	88.44321	33.38874	19.26246	50.61982
FOM-3007	0	26.17903	37.96487	0	10.63358	0	48.80047	14.38361	0	37.96487
FOM-3008	12.84164	52.7308	25.30991	0	10.63358	0	64.54516	23.79966	12.84164	25.30991
FOM-3009	0	39.26854	50.61982	0	10.63358	0	72.69708	19.17815	0	50.61982
FOM-3010	26.1862	39.48247	12.15204	0	5.316789	0	62.84322	14.91266	26.1862	12.15204
NTBA	26.31014	0	12.65496	0	5.316789	0	11.4461	4.794537	20.77121	12.65496
Irgacure2959	19.44853	13.21376	29.82892	5.749512	4.736863	5.749512	34.81111	0	24.20546	24.26547

Monomer	TPSA	EState_VSA1	EState_VSA10	EState_VSA2	EState_VSA3	EState_VSA4	EState_VSA5	EState_VSA6	EState_VSA7	EState_VSA8
FOM-3006	144.09	11.4461	19.17815	37.54219	58.71738	6.07602	18.22806	0	0	47.5829
FOM-3007	78.51	0	14.38361	17.72154	26.17903	12.15204	10.97593	0	0	30.37038
FOM-3008	85.89	0	9.589074	11.81436	52.7308	12.84164	12.15204	0	0	23.79145
FOM-3009	98.82	0	19.17815	62.89726	0	12.15204	21.95186	0	0	36.94932
FOM-3010	86.3	10.11813	17.76508	11.66003	24.9595	23.99337	0	6.923737	14.09534	11.89572
NTBA	29.1	0	4.794537	11.4461	0	0	6.07602	0	20.77121	11.89572
Irgacure2959	66.76	5.601051	9.901065	18.99701	11.31296	0	13.84747	24.26547	0	0

Monomer	EState_VSA9	VSA_EState1	VSA_EState2	VSA_EState3	VSA_EState4	VSA_EState5	VSA_EState6	VSA_EState7	VSA_EState8	VSA_EState9
FOM-3006	14.21059	17.35834	45.90353	10.83913	-1.00956	-1.21352	0	6.385527	16.1532	0
FOM-3007	0	0	34.92471	5.126868	0	-0.86331	0	3.498593	11.31314	0
FOM-3008	14.21059	16.04312	21.69983	5.337454	0	-0.33996	0	3.998361	11.09453	0
FOM-3009	0	0	49.12371	5.155519	0	-1.30326	0	4.613057	15.07764	0
FOM-3010	0	32.17047	11.26999	2.772735	0.497752	-0.41875	0	1.942835	7.512341	0.002632
NTBA	0	0	10.59826	2.711806	-0.14755	-0.12269	0	1.269097	9.107731	0
Irgacure2959	9.84339	5.1517	11.66073	18.10137	-0.93347	0.252365	6.447707	0	3.069593	0

Monomer	FractionCSP3	HeavyAtom Count	NHOH Count	NOCount	NumAromatic Carbocycles	NumArom aticRings	NumH Acceptors	NumH Donors	Num Heteroatoms	NumRotatabl eBonds
FOM-3006	0.52	36	4	11	0	0	7	4	11	23
FOM-3007	0.307692	19	2	6	0	0	3	2	6	9
FOM-3008	0.625	23	2	7	0	0	5	2	7	16

FOM-3009	0.333333	26	2	8	0	0	4	2	8	13
FOM-3010	0.769231	20	1	6	0	0	4	1	7	10
NTBA	0.571429	9	1	2	0	0	1	1	2	1
Irgacure2959	0.416667	16	2	4	1	1	4	2	4	5

Monomer	RingCount	MolLogP	MolMR	fr_Al_OH	fr_Al_OH_ noTert	fr_C_O	fr_C_O_ noCOO	fr_NH0	fr_NH1	fr_amide
FOM-3006	0	0.1541	136.9988	0	0	4	4	0	4	4
FOM-3007	0	-0.3946	73.1904	0	0	3	3	1	2	3
FOM-3008	0	0.4208	88.1044	0	0	2	2	0	2	2
FOM-3009	0	-0.38	99.9674	0	0	4	4	2	2	4
FOM-3010	0	0.4706	78.0813	0	0	1	1	1	1	1
NTBA	0	1.0871	38.0927	0	0	1	1	0	1	1
Irgacure2959	1	1.0113	59.6511	2	1	1	1	0	0	0

Monomer	fr_benzene	fr_ether	fr_ketone	fr_ketone_ Topliss	fr_quatN	fr_unbrch_ alkane
FOM-3006	0	3	0	0	0	9
FOM-3007	0	0	0	0	0	0
FOM-3008	0	3	0	0	0	12
FOM-3009	0	0	0	0	0	0
FOM-3010	0	0	0	0	1	2
NTBA	0	0	0	0	0	0
Irgacure2959	1	1	1	1	0	0

Table S4. The parameter of each Random Forest classifier model.
This table demonstrates the parameters of random forest classifiers by conducting hyperparameter tuning.

Model		Criteria of contact angle	criterion	max_depth	n_estimators	random_state
1	GBM-RFE	20	gini	10	11	15
		25	gini	10	12	18
		30	entropy	10	12	11
		35	gini	10	10	14
		40	entropy	10	13	10
2	RF-RFE	20	gini	10	13	16
		25	gini	10	19	15
		30	entropy	10	10	17
		35	entropy	10	11	19
		40	gini	10	13	12
3	SVM-RFE	20	gini	10	10	17
		25	gini	10	14	12
		30	gini	10	18	12
		35	gini	10	10	17
		40	gini	10	13	19
4	XGB-RFE	20	entropy	10	11	11
		25	entropy	10	15	18
		30	entropy	10	10	10
		35	gini	10	10	13
		40	gini	10	11	19
5	No RFE	20	gini	10	10	19
		25	gini	10	12	14
		30	entropy	10	17	16
		35	entropy	10	10	17
		40	gini	10	13	13
